# Evaluating de Bruijn Graph Assemblers on 454 Transcriptomic Data

**DOI:** 10.1371/journal.pone.0051188

**Published:** 2012-12-07

**Authors:** Xianwen Ren, Tao Liu, Jie Dong, Lilian Sun, Jian Yang, Yafang Zhu, Qi Jin

**Affiliations:** MOH Key Laboratory of Systems Biology of Pathogens, Institute of Pathogen Biology, Chinese Academy of Medical Sciences & Peking Union Medical College, Beijing, China; The Roslin Institute, University of Edinburgh, United Kingdom

## Abstract

Next generation sequencing (NGS) technologies have greatly changed the landscape of transcriptomic studies of non-model organisms. Since there is no reference genome available, *de novo* assembly methods play key roles in the analysis of these data sets. Because of the huge amount of data generated by NGS technologies for each run, many assemblers, e.g., ABySS, Velvet and Trinity, are developed based on a de Bruijn graph due to its time- and space-efficiency. However, most of these assemblers were developed initially for the Illumina/Solexa platform. The performance of these assemblers on 454 transcriptomic data is unknown. In this study, we evaluated and compared the relative performance of these de Bruijn graph based assemblers on both simulated and real 454 transcriptomic data. The results suggest that Trinity, the Illumina/Solexa-specialized transcriptomic assembler, performs the best among the multiple de Bruijn graph assemblers, comparable to or even outperforming the standard 454 assembler Newbler which is based on the overlap-layout-consensus algorithm. Our evaluation is expected to provide helpful guidance for researchers to choose assemblers when analyzing 454 transcriptomic data.

## Introduction

RNA-Seq, the sequencing of cDNAs with next-generation sequencing (NGS) technologies, allows rapid and comprehensive characterization of transcriptomes [1]–[8]. Particularly, with the development of *de novo* assembly software, *ab initio* identification of full-length transcripts from RNA-Seq data has without a reference become possible [2], [9]. Thus, RNA-Seq with *de novo* assembly is very important both for genome annotation, if genome sequences are available, and for functional genomics studies of non-model organisms whose genomes have not yet been sequenced.

NGS technologies, such as the Roche 454, Illumina/Solexa, and ABI SOLiD platforms, produce millions of short sequence reads that vary in length from tens of base pairs (bps) to ∼1,000 bps. Even though read length increases with advances in NGS technologies, the *de novo* assembly of short reads to produce full-length transcripts remains a problem due to sequencing errors and the complexity of transcriptomes.

Recently, there have been various *de novo* assembly algorithms released, including algorithms for genome assembly, e.g., ABySS [10], ALL-PATHS [11], Euler-sr [12], SOAPdenovo [13], SGA [14], and Velvet [15], and algorithms for transcriptome assembly, e.g., MIRA [16], Trans-ABySS [17], [18], Oases [19], and Trinity [2]. Because of the huge amount of data generated by NGS technologies (especially the Illumina/Solexa platform) for each run, many assemblers, e.g. ABySS, ALL-PATHS, Euler-sr, SOAPdenovo, Velvet, Oases and Trinity, are developed based on de Bruijn graph due to its time- and space-efficiency. These tools provide efficient and effective methods to reconstruct full-length transcripts from RNA-Seq data.

However, most of these tools were initially designed and evaluated on the Illumina/Solexa platform that produces millions of short reads with the same length. Their performance on 454 transcriptomic data has not been addressed to our best knowledge. Roche 454 platform generates fewer but longer reads with various lengths than the Illumina/Solexa platform. And Newbler and MIRA are now still the gold standard assemblers for 454 data. What the 454 sequencing properties impact on these Illumina/Solexa-specialized assemblers and whether there is alternative de Bruijn graph based assembler to Newbler and MIRA is unknown.

Although a series of examinations regarding relative performance of various *de novo* assemblers have been published [20], [21], [22], [23], [24], these were all conducted for genome assembly and considered only one NGS platform. Zhao *et al.* evaluated various assemblers rigorously for transcriptome assembly [25], but they only conducted the comparison on the Illumina/Solexa platform. Mundry *et al.* evaluated multiple assemblers on 454 transcriptomic data by a simulation method [26], but only one de Bruijn graph based assembler (Oases) was included in the evaluation. A relatively more comprehensive assessment of de Bruijn graph based assemblers on 454 transcriptomic data is of a pressing need.

In this study, we critically evaluated several of the leading de Bruijn graph based assemblers on both simulated and real 454 datasets. We compared their performance to that of Newbler and MIRA in terms of various metrics, especially including sensitivity and specificity. We found that some de Bruijn graph based assemblers performed bad on 454 transcriptomic data but some assemblers, especially Trinity [2], performed even better than Newbler and MIRA, providing an alternative solution for researchers to analyze 454 transcriptomic data.

## Results

### Overview of Assemblers and Datasets

ABySS (version 1.3.4) [10], Euler-sr (version 1.1.2) [12], SOAPdenovo and SOAPdenovo-Trans (version 1.05) [13], Velvet (version 1.2.07) [15], Oases (version 0.2.08) [19], and Trinity (version r20120608) [2] were selected to assemble the sequence reads. All of these algorithms are based on the de Bruijn graph [27] but implement it differently. In a de Bruijn graph, a node is defined by a sequence with fixed length k (size of ‘k-mer,’ a predefined parameter in many assembly algorithms). An edge, eij, connects node i to node j if and only if the 3′ k−1 nucleotides of node i exactly match the 5′ k−1 nucleotides of node j and the connection is supported by the data. An assembly corresponds to a set of paths in the de Bruijn graph. Variations among different assembly algorithms rely on the error correction steps before and during the construction of the de Bruijn graph, the criteria used for path calling, and the post-processing steps. When assembling our data sets, we set k equal to 25 (Trinity only accepts k-mer size 25) for all assemblers for uniformity and optimized other parameters to achieve a good assembly (see Materials and Methods for details). Although k = 25 may be unfair for some assemblers and multiple k will greatly increase the identification chance of real transcripts, we used the same k for all the de Bruijn graph assemblers because the same k-mer distribution is important to evaluate how different implementations impact the assembly performances. We also evaluated the performance variation of the same assemblers under different k-mer sizes. We selected Newbler (version 2.6, with option ‘-cdna’) and MIRA (version 3.4) [16] as the gold standard assemblers [26].

**Figure 1:**
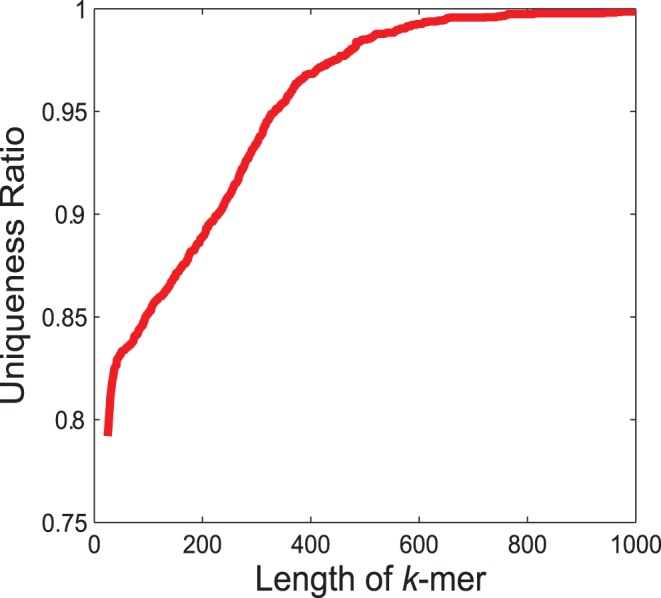
The k-mer uniqueness of *Saccharomyces cerevisiae* transcripts. Fixing the length of k-mer, if one cDNA sequence has no k-mer overlapping with other cDNAs in the data set, then this cDNA sequence is k-mer-unique. The k-mer-uniqueness of a data set is the proportion of k-mer-unique cDNAs in the entire set of cDNAs. In principle, k-mer-uniqueness increases as the length of k-mer increases, and the larger the k-mer-uniqueness is, the easier the *de novo* assembling task.

We used ART (version 1.6.8) [28] to simulate the Roche 454 sequencing data sets based on the full set of *Saccharomyces cerevisiae* cDNA sequences downloaded from Biomart on Dec. 30, 2011 (http://www.biomart.org/biomart/martview/). ART provides a set of simulation tools to generate artificial sequence reads by mimicking the real sequencing process, supporting Roche 454, Illumina/Solexa and Applied Biosystems SPLiD platforms. It has been widely used in the 1000 genomes project (http://www.1000genomes.org/). The error models and read length distributions used in ART are summarized from large number of real data sets. Thus, ART is thought to provide realistic simulation of the true sequencing process. In total, 7,130 *Saccharomyces cerevisiae* cDNA sequences were used in the simulation, and the complexity of these sequences is demonstrated by the plot of length of k-mer against k-mer-uniqueness (Figure 1). Fixing the length of k-mer, if one cDNA sequence has no k-mer overlapping with other cDNAs in the data set, then this cDNA sequence is k-mer-unique. The k-mer-uniqueness of a data set is the proportion of k-mer-unique cDNAs in the entire set of cDNAs. In principle, k-mer-uniqueness increases as the length of k-mer increases, and the larger the k-mer-uniqueness is, the easier the *de novo* assembling task. Figure 1 shows that the k-mer-uniqueness of *Saccharomyces cerevisiae* cDNA sequences approaches 1 as the length of k-mer approaches 1000. When k = 25, the k-mer-uniqueness is ∼0.80, indicating that some cDNAs may not be easily resolved due to the shared k-mers. We simulated three 454 data sets, one with a constant 30-fold coverage for each cDNA and with read length between 37 bps and 385 bps (default read length distribution, totally 1,281,666 reads), the second with varying coverage from 0 to 30 (median: 14, inter-quantile range: 15) for each cDNA and with read length between 37 bps and 385 bps (default read length distribution, totally 605,732 reads), and the third with varying coverage from 0 to 30 (median:15, inter-quantile range: 15) for each cDNA and with read length between 87 bps and 435 bps (customized read length distribution, totally 643,462 reads). The first data set provides sufficient coverage for each cDNA so that the performance of assemblers is evaluated on a relatively easily-solved data set. The second data set provides varying coverage for cDNAs with short read length while the third data set with long read length. Thus, the performance of assemblers on realistic conditions and the impact of read length on assemblers are examined. Here, the varying cDNA coverage summarized the copy numbers of a certain cDNA and the sequencing depth (see Materials and Methods for details).

We also completed the evaluation on a real 454 transcriptomic dataset. We sequenced the transcriptome of *Trichophyton rubrum* by 454 and got 317,624 reads. *Trichophyton rubrum* is a fungus that causes human skin infections worldwide [29], [30], [31], of which the genome has not been completely revealed (Dermatophyte Comparative Sequencing Project, Broad Institute of Harvard and MIT, http://www.broadinstitute.org/) and the transcriptomic catalogue has not been finished. Such a dataset is a practical example showing the relative performance of de Bruijn graph assemblers for transcript reconstruction based on 454 reads without reference genome.

### Evaluation on 30-fold Coverage Simulation Dataset

**Figure 2:**
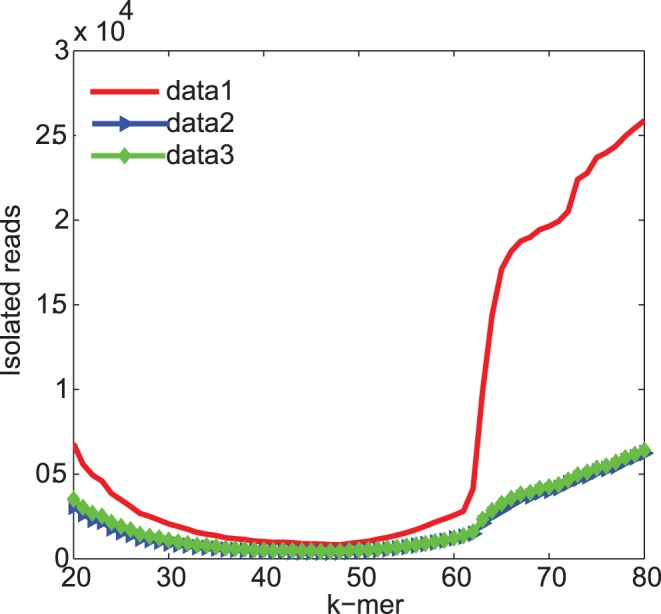
The number of useless reads in the three *Saccharomyces cerevisiae* data sets. Data set 1 was of constant 30-fold coverage. Data set 2 and 3 were of varying coverage. Data set 1 and 2 were generated with the default read length distribution of ART whereas data set 3 was generated with a customized longer read length distribution. If there are repeated k-mers in sequence or all the k-mers are isolated in the de Bruijn graph, then this read is defined as “useless” here.

First, we evaluated the assemblers on the 30-fold coverage simulation dataset. One difficulty of transcriptome assembly is the heterogeneous coverage level compared to genome assembly. We simulated a homogeneous 30-fold coverage dataset to provide a relatively sufficient depth so that genome assemblers assuming homogeneous coverage distribution can also be evaluated justly. The useless reads that have repeated k-mer in sequence and are isolated in the de Bruijn graph of this data set is plotted in Figure 2. It can be seen that when the k-mer size is greater than 60, the proportion of unusable reads increases rapidly.

Standard metrics describing the assembly, e.g. the number of contigs, the longest contig, N50 and N90, were summarized in Table 1. The N50 or N90 value is defined in this manuscript as the contig length L for which half or 90% of the bases in the assembly are in sequences of length N> = L. Although they may be not applicable to transcriptome assembly, we used them to summarize basic characteristics of assemblies. From Table 1, we found that transcriptome assemblers, i.e., Oases, Trinity, Newbler (version 2.6, for transcriptome) and MIRA, generated relatively longer contigs than genome assemblers (ABySS, Euler-sr, SOAPdenovo and Velvet). SOAPdenovo-Trans generated the median-length contigs. The longest contigs of Oases, Trinity, SOAPdenovo-Trans, Newbler and MIRA assemblies were all of more than 11 k bps whereas the longest contigs of ABySS, SOAPdenovo and Velvet were all of fewer than 10 k bps. The N50 values of Oases, Trinity, Newbler and MIRA assemblies were all bigger than 1.7 k while the N50 values of ABySS, SOAPdenovo and Velvet were all smaller than 1.2 k. Euler-sr is an exception of the genome assemblers. It generated contigs whose length distribution was similar to those of transcriptome assemblers.

**Table 1:** Basic characteristics of various assemblies of *Saccharomyces cerevisiae* data set 1 with constant 30-fold coverage.

	ABySS	Euler-sr	SOAPdenovo	SOAPdenovo-Trans	Velvet	Oases	Trinity	Newbler	MIRA
Longest Contig (bps)	9254	14606	3587	11190	9312	14687	11218	14707	14717
#Contigs > = 100 bps	10445	6278	21110	8134	10534	6143	7262	6138	6559
#Contigs > = 500 bps	5568	4792	4795	5397	5902	5130	5451	4872	5008
#Contigs > = 1 k bps	2609	3247	942	3218	2805	3583	3608	3316	3407
N50 (bps)	1021	1804	349	1465	1125	1898	1715	1821	1808
N90 (bps)	164	686	45	487	353	798	639	714	691

Besides these basic metrics, we also evaluated the sensitivity and specificity of these assemblies, which reflect the quality of an assembly. We aligned the assembled contigs to the true transcripts by BLAST (version 2.2.22, with parameters ‘-e 1e-5–F F’) [32] and calculated the sensitivity and specificity indices of each assembly. Sensitivity was defined as the proportion of true transcripts that were reconstructed by an assembly and specificity was defined as the proportion of assembled contigs that can be aligned the true transcripts [26]. If an assembly has both high sensitivity and high specificity, then it is a comprehensive and reliable assembly. The sensitivity and specificity of these assemblers on the 30-fold coverage simulation datasets were summarized in Table 2. It can be found that transcriptome assemblers (Oases, Trinity, Newbler and MIRA, except SOAPdenovo-Trans) outperformed genome assemblers (ABySS, Euler-sr, SOAPdenovo and Velvet). MIRA performed best, with both the highest sensitivity and specificity. Although Oases and Trinity were initially designed for Illumina/Solexa reads, they had comparable performance to Newbler. Especially, Trinity outperformed Newbler for reconstruct 95%-full-length transcripts, with 0.738 sensitivity while Newbler only had 0.560 sensitivity. Trinity also generated the second most reliable contigs, with 0.682 specificity while Newbler only 0.640. Relaxing the contig sequence coverage and the transcript sequence coverage in the alignment (from 95% to 90%, 85% and 80%), Newbler got higher specificity than Trinity but Trinity still had higher sensitivity than Newbler.

**Table 2:** Sensitivity and specificity of various assemblies of *Saccharomyces cerevisiae* data set 1 with constant 30-fold coverage.

SeqCov	Index	ABySS	Euler-sr	SOAPdenovo	SOAPdenovo-Trans	Velvet	Oases	Trinity	Newbler	MIRA
95%	Sensitivity	0.050	0.450	0.067	0.448	0.230	0.539	0.738	0.560	0.811
	Specificity	0.010	0.430	0.006	0.288	0.140	0.647	0.682	0.640	0.818
90%	Sensitivity	0.170	0.640	0.098	0.600	0.310	0.658	0.783	0.700	0.851
	Specificity	0.040	0.620	0.009	0.385	0.180	0.784	0.724	0.800	0.859
85%	Sensitivity	0.290	0.720	0.112	0.647	0.360	0.705	0.800	0.720	0.865
	Specificity	0.080	0.700	0.010	0.414	0.210	0.839	0.740	0.820	0.87
80%	Sensitivity	0.380	0.760	0.128	0.681	0.390	0.732	0.816	0.740	0.875
	Specificity	0.100	0.730	0.011	0.436	0.240	0.871	0.756	0.850	0.879

SeqCov: sequence coverage. When 95%, 90%, 85% and 80% of both the query sequence (contigs) and the subject sequence (true transcripts) were aligned by BLAST (version 2.2.22, with parameters ‘-e 1e–5–F F’), the trancripts were thought to be reconstructed by the respective contigs and the sensitivity and specificity were calculated, respectively.

### Evaluation on Varying Coverage Simulation Dataset

The homogenous coverage simulation dataset provided a baseline comparison of the performance of multiple de Bruijn graph assemblers and the overlap-layout-consensus assemblers Newbler and MIRA. We further generated two datasets with heterogenous coverage to simulate the real transcriptome, one with short read length and the other with long read length. The basic characteristics of the multiple assemblies on the data set with short read length were summarized in Table 3. As the situation of the homogeneous coverage dataset, transcriptome assemblers (Oases, Trinity, Newbler and MIRA, except SOAPdenovo-Trans) generated longer contigs than genome assemblers (ABySS, Euler-sr, SOAPdenovo and Velvet). The longest contigs generated by Oases, Trinity and Newbler all had more than 9 k bps while the longest contigs generated by ABySS, SOAPdenovo and Velvet had fewer than 7 k bps wit Euler-sr an exception. Trinity and MIRA generated the longest contigs with 12251 and 12255 bps, respectively. The N50 values of transcriptome assemblers were also larger than those of genome assemblers. Oases had the largest N50 whereas SOAPdenovo had the smallest.

**Table 3:** Basic characteristics of various assemblies of *Saccharomyces cerevisiae* data set 2 with varying coverage and short read length.

	ABySS	Euler-sr	SOAPdenovo	SOAPdenovo-Trans	Velvet	Oases	Trinity	Newbler	MIRA
Longest Contig (bps)	5836	9197	5584	5144	6689	9116	12251	9208	12255
#Contigs > = 100 bps	10592	8957	16641	10823	8249	6808	7963	6774	8380
#Contigs > = 500 bps	4083	4343	4831	4955	4130	4270	4836	4262	4696
#Contigs > = 1 k bps	1597	2324	1439	2114	1893	2484	2786	2419	2729
N50 (bps)	774	1310	532	943	1057	1521	1471	1477	1474
N90 (bps)	127	323	48	270	310	472	438	442	397

Regarding sensitivity and specificity, MIRA and Trinity outperformed the other assemblers again (Table 4). When the alignment criteria were set to 95% coverage for both query contigs and subject oracle transcripts, MIRA had 0.544 sensitivity and 0.424 specificity. Trinity had 0.440 sensitivity and 0.360 specificity while Newbler had 0.300 sensitivity and 0.260 specificity. The fourth top assembler is Oases, with 0.301 sensitivity and 0.300 specificity. Euler-sr performed best among those genome assemblers.

**Table 4:** Sensitivity and specificity of various assemblies assembled with *Saccharomyces cerevisiae* data set 2 with varying coverage and short read length.

SeqCov	Index	ABySS	Euler-sr	SOAPdenovo	SOAPdenovo-Trans	Velvet	Oases	Trinity	Newbler	MIRA
95%	Sensitivity	0.000	0.220	0.100	0.147	0.150	0.301	0.498	0.300	0.544
	Specificity	0.000	0.140	0.000	0.092	0.090	0.300	0.397	0.260	0.424
90%	Sensitivity	0.100	0.380	0.150	0.273	0.230	0.423	0.616	0.490	0.653
	Specificity	0.000	0.240	0.020	0.170	0.150	0.425	0.488	0.430	0.508
85%	Sensitivity	0.140	0.470	0.200	0.340	0.300	0.485	0.650	0.570	0.687
	Specificity	0.000	0.300	0.030	0.211	0.180	0.487	0.515	0.500	0.533
80%	Sensitivity	0.200	0.530	0.240	0.384	0.350	0.527	0.676	0.610	0.714
	Specificity	0.060	0.340	0.040	0.238	0.220	0.529	0.534	0.530	0.552

SeqCov: sequence coverage. When 95%, 90%, 85% and 80% of both the query sequence (contigs) and the subject sequence (true transcripts) were aligned by BLAST (version 2.2.22, with parameters ‘-e 1e–5–F F’), the transcripts were thought to be reconstructed by the respective contigs and the sensitivity and specificity were calculated, respectively.

We also evaluated the performance of these assemblers on data set 3 in which the read length was longer than that in data set 2. Because Trinity and Oases are Illumina/Solexa-specific transcriptomic assemblers, they may benefit from short read length compared to Newbler and MIRA. Evaluation on longer read length can examine the impact of read length on the performance of these assemblers. The basic characteristics of the assemblies on data set 3 were summarized in Table 5. Trinity generated the longest contig (14654 bps) while Newbler generated the second longest contig (14586 bps). Oases and MIRA generated the third and fourth longest contigs (12242 and 12240 bps, respectively). Oases had the longest N50 and N90 values. The sensitivity and specificity of these assemblies were summarized in Table 6. MIRA still performed best with regards to both sensitivity and specificity. Trinity was the second best assembler and Newbler was the third best. Oases performed the fourth. These results suggest that Trinity is comparable to Newbler and MIRA not only on data set with short reads but also on data set with long reads.

**Table 5:** Basic characteristics of various assemblies of *Saccharomyces cerevisiae* data set 3 with varying coverage and long read length.

	ABySS	Euler-sr	SOAPdenovo	SOAPdenovo-Trans	Velvet	Oases	Trinity	Newbler	MIRA
Longest Contig (bps)	6020	12160	5629	6782	9055	12242	14654	14586	12240
#Contigs > = 100 bps	10693	8075	17135	10995	8411	6815	8043	6824	8443
#Contigs > = 500 bps	4160	3969	4840	5021	4222	4411	4852	4345	4704
#Contigs > = 1 k bps	1631	2144	1365	2118	1934	2562	2815	2497	2713
N50 (bps)	780	1317	508	927	1059	1530	1486	1476	1472
N90 (bps)	125	326	47	269	309	502	435	455	390

**Table 6:** Sensitivity and specificity of various assemblies assembled with *Saccharomyces cerevisiae* data set 3 with varying coverage and long read length.

SeqCov	Index	ABySS	Euler-sr	SOAPdenovo	SOAPdenovo-Trans	Velvet	Oases	Trinity	Newbler	MIRA
95%	Sensitivity	0.020	0.218	0.071	0.152	0.151	0.307	0.508	0.342	0.531
	Specificity	0.006	0.166	0.011	0.077	0.108	0.314	0.397	0.327	0.408
90%	Sensitivity	0.075	0.356	0.142	0.273	0.229	0.438	0.618	0.526	0.641
	Specificity	0.022	0.269	0.022	0.138	0.164	0.442	0.483	0.505	0.495
85%	Sensitivity	0.145	0.445	0.188	0.338	0.283	0.498	0.653	0.592	0.683
	Specificity	0.043	0.335	0.029	0.171	0.202	0.502	0.511	0.567	0.525
80%	Sensitivity	0.212	0.501	0.222	0.382	0.326	0.536	0.677	0.624	0.706
	Specificity	0.062	0.376	0.034	0.193	0.233	0.539	0.529	0.597	0.542

SeqCov: sequence coverage. When 95%, 90%, 85% and 80% of both the query sequence (contigs) and the subject sequence (true transcripts) were aligned by BLAST (version 2.2.22, with parameters ‘-e 1e–5–F F’), the transcripts were thought to be reconstructed by the respective contigs and the sensitivity and specificity were calculated, respectively.

We also checked different k-mer sizes for the same assemblers on data set 3 to show the impact of k-mer size on assemblies. For ABySS, we tested k-mer sizes 25–31. The sensitivity and specificity increased slightly (sensitivity from 0.020 to 0.023, specificity from 0.006 to 0.008, 95% sequence coverage), suggesting that the performance differences between assemblers are not due to k-mer bias. For Euler-sr, we tested 25–27 (the larger k-mer is, the more time the computation needs). The sensitivity increased from 0.218 (k-mer: 25) to 0.229 (k-mer: 26) and then decreased to 0.210 (k-mer: 27). The variation was still much smaller than the differences between assemblers. The small variation of the same assembler under different k-mer sizes was also supported by SOAPdenovo, SOAPdenovo-Trans, Velvet and Oases. Because Trinity only accepts k-mer size 25, the effect of different k-mer sizes was not evaluated on it. Results on data set 1 and 2 also supported small variation between different k-mer sizes of the same assemblers.

The simulated data sets also allowed us to check the chimeric contigs in these assemblies. By aligning the contigs to the true transcripts and counting how many true transcripts were aligned to one contig (E-value <1e-5, contig coverage >10%), we found that Newbler reported the fewest chimeric contigs on both data set 2 (557 chimeric contigs out of 6936 total) and 3 (583 chimeric contigs out of 6968 total). The second assembler with fewest chimeric contigs was Oases (568 chimeric contigs out of 6808 total on data set 2 and 604 chimeric contigs out of 6815 total on data set 3). The ratios for all assemblers are about 8% (Table 7).

**Table 7:** Chimeras identified in the different assemblies on *Saccharomyces cerevisiae* data set 2 and 3.

	ABySS	Euler-sr	SOAPdenovo	SOAPdenovo-Trans	Velvet	Oases	Trinity	Newbler	MIRA
#Contigs in data set 2	22106	9918	41017	13194	9191	6808	8373	6936	8512
#Chimera in data set 2	908	690	1206	804	645	568	794	557	692
#Contigs in data set 3	22707	8900	43845	13261	9365	6815	8471	6968	8568
#Chimera in data set 3	1135	672	1485	858	741	604	784	583	672

If a contig has two subsequences (at least 10% of the contig length) that aligned to two different true transcripts (BLASTN v2.2.22, parameters: -e 1e–5–F F), then this contig is thought to be a chimera.

### Evaluation on Real *Trichophyton rubrum* Dataset

We further examined the performance of the assemblers on a real non-model organism dataset to evaluate the practical power of the assemblers. We sequenced the transcriptome of *Trichophyton rubrum* by 454 and obtained 317,624 reads with various lengths (from 40 bps to 1185 bps). Then, the assemblers were applied to this dataset to reconstruct transcripts of *Trichophyton rubrum*. The basic metrics of the assemblies were summarized in Table 8. Oases, Trinity and Newbler generated longer contigs than genome assemblers ABySS, Euler-sr, SOAPdenovo and Velvet. Newbler generated the longest contig (22,223 bps). Trinity generated the second longest contig (17023 bps).

**Table 8:** Basic characteristics of various assemblies of real *Trichophyton rubrum* data.

	ABySS	Euler-sr	SOAPdenovo	Velvet	Oases	Trinity	Newbler	MIRA
Longest Contig (bps)	3986	6326	4349	2802	10856	17023	22223	7950
#Contigs > = 100 bps	34779	37100	65842	67119	15839	18800	13294	26674
#Contigs > = 500 bps	10534	13479	16166	9263	8215	15069	9652	22063
#Contigs > = 1 k bps	3139	3965	3699	1230	3561	9645	4858	8368
N50 (bps)	531	692	474	324	1071	2188	1233	1115
N90 (bps)	48	207	84	83	369	690	552	526

Because SOAPdenovo-Trans did not produce results, the corresponding indices are not available.

Because of the absence of the true transcripts of *Trichophyton rubrum,* unlike the situations in simulation, a difficulty occurred in evaluating the sensitivity and specificity of assemblers. Thus, we used the NCBI non-redundant protein database (NR) as reference to examine how many of the assembled contigs encode open reading frames (ORFs) that can be aligned to known proteins. The assumption of this evaluation is the conservation of proteins among organisms. We found that Trinity and Oases can assemble more contigs encoding ORFs that can be aligned to known proteins in NR. Setting the query coverage and subject coverage in the alignment both more than 95%, Trinity had 1,466 (11%) contigs encoding ORFs aligned to proteins in NR. 847 out of the 1,466 contigs encode ORFs that can be aligned globally to the full-length proteins in NR. Oases had 1439 (9%) contigs encoding ORFs aligned to proteins in NR. 920 out of 1439 aligned globally. MIRA had 835 (4%) contigs encoding ORFs aligned to proteins in NR. 494 out of 835 aligned globally. Newbler had 763 (6%) contigs encoding ORFs aligned to protein in NR with 95% as the alignment criteria. 624 encode ORFs that can be aligned globally to the full-length proteins in NR. If the alignment criteria were set to 90% for both query coverage and subject coverage, then Trinity and Oases had 2055 (15%) and 1884 (12%) aligned contigs whereas MIRA and Newbler had 1307 (7%) and 919 (7%) aligned contigs, further suggesting the excellent performance of Trinity.

## Discussion

The NGS technologies have greatly changed the landscape of transcriptome research of non-model organisms. Without reference genome, the NGS technologies can generate many but short sequence reads of the transcriptome of non-model organisms. With the *de novo* assemblers, these short reads can be assembled into longer contigs which approximates transcripts or something similar. However, the *de novo* assembly of short reads to long transcripts is a challenging task due to short repeated sequences, sequencing errors, and other reasons. Now there have been many *de novo* assemblers released to facilitate researchers’ usage. Among the numerous assemblers, de Bruijn graph based assemblers and overlap-layout-consensus based assemblers dominate the market. Because of the huge size of the NGS output and the time- and space-efficacy of de Bruijn graph based assemblers, de Bruijn graph based assemblers get more and more popular. However, most of the current de Bruijn graph based assemblers were initially designed and tested on the Illumina/Solexa platform. There are fewer de Bruijn graph based assemblers for Roche 454 platform. The gold standard assemblers on 454 are Newbler and MIRA, two overlap-layout-consensus based assemblers. It is still unclear whether the Illumina/Solexa-specialized de Bruijn graph assemblers are applicable on the 454 platform and whether there is alternative to or better assemblers than Newbler and MIRA for 454 transcriptomic analysis. In this study, we conducted such an evaluation and found that those Illumina/Solexa-specialized de Bruijn graph assemblers performed well on 454 transcriptomic data. Especially, the transcriptome-specialized assembler, Trinity, is comparable to or even better than the gold standard 454 assemblers, Newbler and MIRA. Our results on both simulated and real datasets suggested that Trinity had both high sensitivity and high specificity, providing a good choice for researchers analyzing 454 transcriptome data.

In our evaluation, all assemblers except Newbler and MIRA are based on de Bruijn graphs. Setting the same k-mer length, we rigorously compared the performance of these assemblers. The results suggested big performance variations, suggesting the big impact of the implementation details of the same algorithm on the transcript reconstruction. First, Oases and Velvet provide an example that different assembly assumptions greatly influence the performance. With the same input and k-mer, Oases wrapped the output of Velvet but significantly outperformed Velvet in reconstructing transcripts. Velvet was initially designed for genome assembly, so some assumptions based on genome sequences were made and encoded in the implementations. Oases was designed for transcriptome assembly and thus broke the genome assumption and got better performance. Second, Euler-sr provides an excellent example that different implementations can also result in significant performance variations even though it had the same input, the same data structure, the same k and the same design aim (genome assembly) as ABySS, SOAPdenovo and Velvet. Among the four genome assembly tools, only Euler-sr had comparable performance to those transcriptome-aimed assemblers. Third, the disparity between Oases and Trinity suggested that implementation differences within transcriptome assembly aimed tools can also result in big performance differences.

Many studies have suggested that multiple k strategy, i.e. constructing different assemblies with multiple k and then merging the multiple assemblies by clustering methods, can greatly improve the sensitivity of the final merged assembly [17], [19], [25]. This strategy is very useful in practical application to reconstruct as many transcripts as possible. But the cost of sensitivity improvement is the decrease of specificity because many redundant/overlapped contigs were generated. Although clustering methods can reduce certain redundancy, multiple k strategy generally generates much more contigs than real transcripts [17], [19], [25]. Moreover, the 5′ and 3′ boundary problem of the reconstructed transcripts cannot be solved by clustering methods, resulting in many partially reconstructed transcripts. A promising method to utilize multiple k-mer distribution information underlying the input data maybe lies in the assembly construction process, just like what IDBA does [33], [34], [35]. Evaluation of multiple k strategy on 454 transcriptomic data is out of the scope of our current study and will be finished in future.

The size and complexity of an organism’s transcriptome may also an important factor influencing the performance of various assemblers. In our evaluations, MIRA performed best on all the data sets of *Saccharomyces cerevisiae*. But on the data set of *Trichophyton rubrum*, a much bigger and more complex fungus, MIRA reported fewer contigs that can align to the NCBI NR database than Trinity and Oases. This may be because of the high efficacy of de Bruijn graph assemblers to handle large data set. A rigorous evaluation of the impact of the transcriptome complexity on performance of assemblers is expected in future.

### Conclusions

The overlap-layout-consensus based assemblers, Newbler and MIRA, are still the gold-standard assemblers for 454 transcriptomic data now. We evaluated a series of initially Illumina/Solex-oriented de Bruijn graph based assemblers on both simulated and real 454 transcriptomic datasets, and found that 1) MIRA performed best with regarding both sensitivity and specificity; 2) Trinity is comparable to or even outperformed Newbler, providing an alternative solution for reconstructing full-length transcripts from 454 reads; and 3) all assemblers generate about 8% chimeric contigs, reminding scientists carefully interpreting the results.

## Materials and Methods

### Ethics Statement

*Trichophyton rubrum* (strain BMU01672) used in this study was isolated from nail scraps of a patient suffering from tinea unguium. The participant provided his written informed consent and the procedure was approved by the ethics committee of Institute of Pathogen Biology, Chinese Academy of Medical Sciences & Peking Union Medical College.

### Simulation of Saccharomyces Cerevisiae Dataset

The full-length sequences of 7,130 *Saccharomyces cerevisiae* cDNAs were downloaded from Biomart on Dec. 30, 2011 (http://www.biomart.org/biomart/martview/), and ART (version 1.6.8) [28] was utilized to simulate Roche 454 and Illumina/Solexa reads with 30-fold homogeneous coverage and heterogeneous coverage between 0 and 30. Given a *Saccharomyces cerevisiae* cDNA sequence, a coverage value between 0 and 30 was randomly assigned and then ART was applied to simulate the 454 reads with the coverage. The product of the copy number of a transcript and the sequencing depth forms the coverage of the transcript. Varying coverage simulated the copy number of transcripts if the sequencing depth is a constant. The default read length distribution of Roche 454 reads was based on the distribution of a large number of real data sets summarized by ART. The customized long read length distribution was based on the default read length distribution except that each read length number was added 50 bps additionally. The sequencing error profiles for Roche 454 were based on those provided by ART. The simulated data sets were direct inputs of the assemblers, without any preprocessing step, e.g. error correction.

### Generation of Real Trichophyton Rubrum Transcriptome Dataset

The *Trichophyton rubrum* isolate was maintained on potato glucose agar (Difco) at 28°C for 2 weeks to produce conidia. Conidia were washed away from the surface of the agar by sterile double-distilled water and filtered twice through a 70-µm nylon filter. The conidia suspension was then transferred into Sabouraud liquid medium (containing 49 g glucose and 10 g Difco Bacto peptone in 1 L distilled water) and cultured with constant shaking (200 rpm, Innova 4230 Refrigerated Incubator Shaker, New Brunswick Scientific) at 28°C for 1 week. Hyphae of *Trichophyton rubrum* collected from the Sabouraud liquid medium was ground into powder with a mortar and pestle in liquid nitrogen to facilitate cell disruption. Total RNA was isolated using the QIAGEN RNeasy® Plant Mini Kit (QIAGEN, Inc., Valencia, CA) and Poly(A)+ mRNA was purified with the Oligotex mRNA Mini Kit (QIAGEN), according to the manufacturer’s instructions. Single- and double-strand synthesis was conducted using the Superscript Double-Strand cDNA Synthesis Kit (Invitrogen). The quality of cDNA was checked on 1.5% agarose gels and an Agilent 2100 Bioanalyzer. Preparation of transcription libraries for sequencing on 454 and the Illumina GA2 platforms was carried out according to the manufacturer’s standard protocol. Briefly, for 454 sequencing, an approximately 5-µg cDNA sample was fractionated into 300–500-bps fragments and subsequently blunted. Short adaptors were then ligated prior to amplification and sequencing. Finally, one sequencing run was performed using the method described by Margulies et al. [36]. The raw data were deposited to the NCBI Sequence Reads Archive (SRA) with accession number SRA050365.1. The real data set was direct inputs of the assemblers, without any preprocessing step, e.g. error correction.

### Parameter Settings of the Assemblers

ABySS (version 1.3.4) [10], Euler-sr (version 1.1.2) [12], SOAPdenovo and SOAPdenovo-Trans (version 1.05) [13], Velvet (version 1.2.07) [15], Oases (version 0.2.08) [19], Trinity (version r20120608) [2], Newbler (version 2.6) and MIRA (version 3.4) were included in this evaluation. For ABySS, Euler-sr, SOAPdenvo, SOAPdenovo-Trans and Oases, except the k-mer size, the other applied parameters were the default values. For Velvet, the ‘-cov_cutoff’ was set to 4. For Trinity, the minimal contig length was set to 25 to include as many contigs as possible. For Newbler, ‘-cdna’ was used. For MIRA, ‘–job = denovo, est, accurate, 454 -fasta -MI:sonfs = no –notraceinfo’ were used to accomplish the assemblies.

For the simulated data sets, the contigs were aligned to the true transcripts downloaded from Biomart (http://www.biomart.org/biomart/martview/) on on Dec. 30, 2011 by the NCBI BLAST software (version 2.2.22) with parameters ‘-p blastn –e 1e–5–F F’. For the real data set, the ORFs encoded in the contigs were first identified by the getorf software in the EMBOSS software package () and then were aligned to the NCBI NR database by the NCBI BLAST software (version 2.2.22) with parameters ‘-p blastp –e 1e-5–F F’.

## Supporting Information

Figure S1 **Trichophyton_rubrum.mRNA.454.trinity20120608.contigs.fasta Contigs assembled by Trinity20120608 based on the 454 reads of** ***Trichophyton rubrum*** **transcriptome.**
(FASTA)

Figure S2 **Trichophyton_rubrum.mRNA.454.abyss1.3.4.k25.contigs.fasta Contigs assembled by ABySS (v1.3.4) based on the 454 reads of** ***Trichophyton rubrum*** **transcriptome.**
(FASTA)

Figure S3 **Trichophyton_rubrum.mRNA.454.euler-sr.1.1.2.k25.contigs.fasta Contigs assembled by Euler-sr (v1.1.2) based on the 454 reads of** ***Trichophyton rubrum*** **transcriptome.**
(FASTA)

Figure S4 **Trichophyton_rubrum.mRNA.454.mira3.4.contigs.fasta Contigs assembled by MIRA (v3.4) based on the 454 reads of** ***Trichophyton rubrum*** **transcriptome.**
(FASTA)

Figure S5 **Trichophyton_rubrum.mRNA.454.oases0.2.k25.contigs.fasta Contigs assembled by Oases (v0.2.0.8) based on the 454 reads of** ***Trichophyton rubrum*** **transcriptome.**
(FASTA)

Figure S6 **Trichophyton_rubrum.mRNA.454.soapdenovo1.05.k25.contigs.fasta Contigs assembled by SOAPdenovo (v1.05) based on the 454 reads of** ***Trichophyton rubrum*** **transcriptome.**
(FASTA)

Figure S7 **Trichophyton_rubrum.mRNA.454.velvet1.2.07.k25.contigs.fasta Contigs assembled by Velvet (v1.2.07) based on the 454 reads of** ***Trichophyton rubrum*** **transcriptome.**
(FASTA)

Figure S8 **Tricophyton_rubrum.mRNA.454.newbler2.6.contigs.fasta Contigs assembled by Newbler (v2.6) based on the 454 reads of** ***Trichophyton rubrum*** **transcriptome.**
(FASTA)
